# iTRAQ Proteomic Analysis of Interactions Between 20E and Phospholipase C in *Apolygus lucorum* (Meyer-Dür)

**DOI:** 10.3389/fphys.2022.845087

**Published:** 2022-02-18

**Authors:** Yong-An Tan, Xu-Dong Zhao, Jing Zhao, Keyan Zhu-Salzman, Qin-Qin Ji, Liu-Bin Xiao, De-Jun Hao

**Affiliations:** ^1^Institute of Plant Protection, Jiangsu Academy of Agricultural Sciences, Nanjing, China; ^2^College of Forestry, Nanjing Forestry University, Nanjing, China; ^3^Department of Entomology, College of Agriculture and Life Sciences, Texas A&M University, College Station, TX, United States; ^4^Taizhou Customs of the People’s Republic of China, Taizhou, China

**Keywords:** *Apolygus lucorum*, 20-hydroxyecdysone, phospholipase C, iTRAQ, proteomics, Western blot

## Abstract

Polyphagous *Apolygus lucorum* has become the dominant insect in *Bacillus thuringiensis* (Bt) cotton fields. Hormone 20-hydroxyecdysone (20E) regulates multiple insect development and physiology events. 20E responses are controlled by pathways triggered by phospholipase C (PLC)-associated proteins. However, 20E-modulated genes and related proteins that can be affected by PLC still remain unknown. Here, isobaric tag for relative and absolute quantitation (iTRAQ) and immunoblotting techniques were used to compare differentially expressed proteins (DEPs) in *A. lucorum* in response to the treatment of 20E and the PLC inhibitor U73122 as well as their combination. A total of 1,624 non-redundant proteins and 97, 248, 266 DEPs were identified in the 20E/control, U73122/control, and 20E + U73122/control groups, respectively. Only 8 DEPs, including pathogenesis-related protein 5-like, cuticle protein 19.8, trans-sialidase, larval cuticle protein A2B-like, cathepsin L1, hemolymph juvenile hormone-binding protein, ATP-dependent RNA helicase p62-like, and myosin-9 isoform X1, were detected in all three groups. Kyoto Encyclopedia of Genes and Genomes (KEGG) enrichment analysis showed that the DEPs were involved in diverse signaling pathways. The results were validated by immunoblotting, which highlighted the reliability of proteomics analysis. These findings provided novel insights into the function of PLC in 20E signaling pathway in *A. lucorum*.

## Introduction

*Apolygus lucorum* (Meyer-Dür) is an agricultural pest found in northern China, damaging more than 200 plant species (Lu and Wu, 2011; Pan et al., 2013). Meyer-Dür adults have high dispersal ability and severely threaten the cotton, and many fruit and vegetable industries. From late 1990s to early 2000s, Bt (*Bacillus thuringiensis*), cotton adoption has effectively curtailed *Helicoverpa armigera* (Hübner), resulting in a reduction of insecticide sprays applied in cotton fields and a subsequent outbreak of mirid bugs (including *A. lucorum*) in many crops (Lu et al., 2010). However, since 2010, the cotton-planted areas have decreased sharply, with the expansion of maize and some other crops, such as vegetables, soybeans, and peanuts. The *A. lucorum* species experiences little pressure from natural enemies (Luo et al., 2014; Li et al., 2017). Moreover, a significant increase in the availability of host plants might have a pronounced effect on *A. lucorum* densities in the local region (Lu and Wu, 2008). Besides, extensive use of insecticides could lead to *A. lucorum* resistance, arousing environmental concerns (Lu et al., 2007). Understanding the regulatory mechanisms underlying insect growth and development is crucial for scientific prevention, control, and integrated management of pests. Consequently, there is an urgent need to develop new pest management strategies.

Insect steroid ecdysone and its active metabolite 20-hydroxyecdysone (20E) coordinate multiple insect development and physiology events, such as caste determination, diapause, and longevity (Tatun et al., 2018). As soluble compounds, steroid hormones can easily diffuse into the cell nucleus *via* the cytoplasmic membrane and interact with nuclear steroid hormone receptors to substantially modulate gene expression (Thompson, 1995). The two nuclear hormone receptors of ecdysone, including the ecdysone receptor (EcR) and ultraspiracle (USP), mediate the function of 20E (Fahrbach et al., 2012). Meanwhile, steroid hormones trigger non-genomic signaling *via* the cytoplasmic membrane (Falkenstein et al., 2000; Meldrum, 2007; Sandén et al., 2011). More recently, Phospholipase C (PLC) has been considered as a regulatory enzyme significantly contributing to modulate the non-genomic 20E signaling pathway, mostly by hydrolyzing the polar head groups in inositol-containing membrane phospholipids (Cai et al., 2014; Liu et al., 2014a). For instance, cell apoptosis in silkworm anterior silk glands is induced by 20E-associated GPCR (G protein-coupled receptors)-PLC-IP_3_ (inositol triphosphate)-Ca^2+^-PKC (Protein Kinase C; Iga et al., 2007; Manaboon et al., 2009). In *H. armigera*, through GPCR-PLC-Ca^2+^ signaling, 20E induces a rapid phosphorylation of cyclin-dependent kinase 10 (CDK10) to promote gene transcription (Liu et al., 2014a). PLC substantially affects larva development and pupation, and 20E upregulates PLC during molting and metamorphosis. Mediated by ecdysone-responsible G (ErGPCR), G protein alpha q (Ga_q_), and Src kinases, 20E readily triggers tyrosine phosphorylation in the SH2 domain of PLC, promoting its migration toward the cytoplasmic membrane. Through PLC/Ca^2+^ signaling, 20E induces ecdysone response element (EcRE) transcriptional activity by modulating PKC phosphorylation at Ser 21, which is essential for its interaction with EcRE (Liu et al., 2014a; Jing et al., 2015).

One previous study found that 20E could regulate soluble trehalase (*AlTre-1*) with the help of *AlPLCγ*, altering vitellogenin (Al*Vg*) expression and ovary development, and facilitating female reproduction in *A. lucorum*. In contrast, treatment with the PLC inhibitor (U73122) significantly downregulated the genes involved in 20E signaling, ultimately leading to delayed growth and development and reduced fecundity of *A. lucorum*. This suggested that PLC participated in the non-genomic 20E signaling pathway and regulated the growth and development of *A. lucorum* (Tan et al., 2021). Currently, differential proteomics can help efficiently identify proteins with physiological significance. Most recently, isobaric tags for relative and absolute quantitation (iTRAQ) were developed to simultaneously assess 4–8 distinct specimens by MS/MS, thereby increasing throughput and reducing assay-related errors (Ross et al., 2004; Ye et al., 2010). Moreover, this novel method is sensitive, automated, and multi-dimensional (Caubet et al., 2010). Herein, iTRAQ was utilized to investigate differentially enriched proteins and related signaling pathways in *A. lucorum* in response to the treatments with 20E, U73122, and their combination. The data might lay the foundation for an improved understanding of the nymphal responses of *A. lucorum* involving 20E and PLC signaling.

## Materials and Methods

### Insect Culture

*Apolygus lucorum* was collected from *Vicia faba* grown in fields in Yancheng (33.110 N, 120.250E), Jiangsu Province, China, from July to August 2018, and reared on *Phaseolus vulgaris* in an incubator at 25 ± 1°C with 70 ± 5% humidity under a 14- to 10-h light/dark cycle.

### Sample Preparation

About 24 h after molting, 600 third instar nymphs were collected into plastic cases with green beans. Midguts from early wandering third instar nymphs were dissected in Ringer’s solution, and pre-incubated in a 12-well tissue culture plate with 500 μl per well of Grace’s insect tissue culture medium for 30 min, with or without 50 μM U73211 (Sigma, St. Louis, MO, United States), followed by incubation with 1.0 μM 20E (Sigma, St Louis, MO, United States) or distilled water for 12 h (Tan et al., 2021). The assay consisted of four experimental treatments: (1) 20E and U73122; (2) 20E; (3) U73122; and (4) CK, the negative control (treatment with distilled water). Midguts were then homogenized, followed by centrifugation (16,000 × *g*, 10 min).

### Protein Extraction and Digestion

Specimens were pulverized in liquid nitrogen and treated with 0.5 ml lysis buffer (6 M urea, 2 M thiourea, 4% CHAPS, 40 mM Tris–HCl, 5 mM EDTA, 1 mM PMSF, and 10 mM DTT; pH 8.0). Supernatants were collected after sonication (5 min at 4°C) and centrifugation (18,000 × *g*, 4°C; 15 min). Protein concentrations were quantified by Bradford assay. According to the FASP method (Tang et al., 2017), protein (100 μg) digestion was performed in 0.1 M tetraethylammonium bromide (TEAB) buffer containing trypsin (Promega, Madison, WI, United States). Two biological replicates with 300 mixed midguts form third instar nymphs, respectively, were used for iTRAQ analysis.

### iTRAQ Labeling

After trypsin digestion, specimen lysates (200 μg) were added to buffered urea (200 μl 8 M urea in 150 mM Tris-HCl pH 8.0) and concentrated by centrifugation (15 min, 14,000 × *g*) using 10 kDa diafiltration tubes. The concentrated samples were incubated at ambient in buffered urea with 100 μl 50 mM iodoacetamide (Bio-Rad, 163-2109, Hercules, CA, United States) and concentrated as described above. After two washes with 100 μl buffered urea and centrifugation (10 min, 14,000 × *g*), the specimens were further resuspended with 100 μl of dissolution buffer (AB SCIEX, Foster City, CA, United States) and centrifuged as above. Pellets in 40 μl dissolution buffer were subjected to digestion with 2 μg trypsin overnight at 37°C, followed by vacuum drying. Further sample processing with the 8-plex iTRAQ reagent (AB SCIEX) was carried out as directed by the manufacturer. Treated specimens were labeled with iTRAQ reagents 113–115 and 116–118 (1 h, ambient), respectively. After vacuum drying, fractionation was carried out by high pH reverse phase chromatography (Hp-RPC).

### Fractionation by Hp-RPC

A Shimadzu LC-20AB HPLC Pump system was used for Hp-RPC. The iTRAQ-labeled peptide mixture was added to 0.5 ml buffer A (20 mM NH_4_HCO_2_ in 2% acetonitrile, pH 10), followed by injection into a Gemini-NX μM C18 column (110 Å, 250 × 4.6 mm; Phenomenex, Guangzhou, China). Elution was carried out at 1 ml/min initially with buffer A for 10 min, then with 5%–30% buffer B (20 mM NH_4_HCO_2_ in 98% acetonitrile, pH 10) for 15 min, followed by 30%–80% buffer B in buffer A for 3 min. Detection was performed at 214 nm, with fraction collection at 1-min interval. Finally, the eluates were pooled into 10 fractions for vacuum drying.

### LC-ESI-MS/MS

Various fractions were resuspended in certain volume of buffer A (5% acetonitrile and 0.1% formic acid in water) and centrifuged (18,000 × *g*, 10 min). The final peptide content was adjusted to approximately 0.5 μg/μl in all fractions. After loading 8 μl of each sample at 3 μl/min on an Eksigent 425 2D HPLC system (AB SCIEX), the peptides were eluted onto an in-house packed analytical C18 column (internal diameter, 75 μm). A 45-min gradient was initially applied at 0.3 μl/min with 8%–25% buffer B (84% acetonitrile and 0.1% formic acid), followed by a 10-min linear gradient with 80% buffer B, which was maintained for 5 min.

A TripleTOF 5,600 system was utilized to acquire data, with an ion spray voltage at 2.3 kV, curtain and ion source gases at 30 and 6 PSI, respectively, and the chamber at 150°C. MS was carried out in the high-resolution mode (>30,000 fwhm) for TOF MS scanning, with a *m/z* of 350–1800 Da. For information-dependent data acquisition (IDA), the scan was performed for 50 ms, collecting up to 30 productions exceeding the cutoff of 200 counts/s with 2+ to 4+ charge.

### iTRAQ Protein Assessment

Raw data (*.wiff and *.wiff.scan) were analyzed with ProteinPilot 5.0 (AB SCIEX) against the *A. lucorum* transcriptome database. Search indices were: Specimen Type, iTRAQ 8plex (Peptide Labeled); Cysteine alkylation, iodoacetamide; Digestion, trypsin. The FDR (false discovery rate) analysis was performed routinely for identification. Differentially expressed proteins (DEPs) were considered to have a 2-fold change cutoff.

### Bioinformatics Analysis

Two annotation databases, including Gene Ontology (GO; Ashburner et al., 2000) and Kyoto Encyclopedia of Genes and Genomes (KEGG; Ogata et al., 1999), were utilized for annotating and grouping DEPs. The GO term/numbers of DEPs were retrieved with the Blast2GO software,^1^ and DEPs were further classified with WEGO 2.0, a web tool for analyzing and plotting GO annotations (Ye et al., 2018). As for KEGG pathway analysis, the protein sequences of DEPs were uploaded onto the KEGG Automatic Annotation Server (Moriya et al., 2007) to compute and group the DEP-integrated signaling pathways.

### Protein Analysis

Immunoblotting analysis was performed to validate iTRAQ data. In brief, proteins were extracted from the treated midguts with Tissue Protein Extraction Reagent Kit (ZoonBio) as directed by the manufacturer and quantified by the bicinchoninic acid (BCA) assay (ZoonBio). Equal amounts of total proteins were separated on 10% SDS-PAGE and electro-transferred onto nitrocellulose membranes (Bio-Rad). After blocking with 5% skim milk in Tris-buffered saline with 0.05% Tween 20 for 1 h at 37°C, the membranes were incubated with primary antibodies against trans-sialidase, cuticle protein 19.8, pathogenesis-related protein 5, guanine nucleotide-binding protein, and ras-related protein 1, respectively for 1 h at 37°C, followed by incubating with horseradish peroxidase (HRP)-linked secondary antibody (ZoonBio) for 1 h at ambient temperature. Luminescent signals generated by Enhanced Chemiluminescence Advance Kit (GE Healthcare, PA, United States) were quantified by densitometry with ImageJ.^2^ Relative protein amounts of trans-sialidase, cuticle protein 19.8, pathogenesis-related protein 5, guanine nucleotide-binding protein, and ras-related protein 1 were normalized against β-actin that was recognized with mouse anti-β-actin antibody (ZoonBio).

## Results

### Protein Profiling and Identification of Differentially Expressed Proteins

The iTRAQ approach was employed to perform comparative analysis among the three groups to obtain a global view of proteome alteration in *A. lucorum* nymphs in response to 20E, U73122, and 20E + U73122. A total of 1,624 non-redundant proteins were identified (Supplementary Table S1). Based on the regulation cutoff of 2.0-fold (*p* < 0.05), 97 (20 upregulated and 77 downregulated), 248 (42 upregulated and 206 downregulated), and 266 (57 upregulated and 209 downregulated) DEPs were identified in the 20E/control, U73122/control and 20E + U73122/control groups, respectively (Supplementary Table S2). Interestingly, the overall number of downregulated proteins whereas higher than that of upregulated ones, and DEPs were significantly less abundant in the 20E/control group compared to the other two groups, in which the numbers of DEPs were comparable. In addition, all identified cuticular proteins were upregulated to varying degrees after 20E treatment (Table 1), such as larval cuticle protein A2B-like, pupal cuticle protein C1B-like, cuticle protein 19.8, and cuticle protein 19-like. Remarkably, 12 cuticular proteins and their homologous molecules were downregulated after U73122 treatment, while 7 cuticular proteins and their homologous molecules were downregulated by 20E + U73122 (Tables 2 and 3).

**Table 1:** Cuticle proteins and homologies in 20E-treated *A. lucorum*.

Protein	Blast similarity mean (%)	Peptides (95%)	*p*-value	Ratio (average)	Accession
Larval cuticle protein A2B-like	95.12	293	0.021508288	2.780555367	CL1466.Contig2
Pupal cuticle protein C1B-like	94.02	365	0.0361844	2.660523891	CL31.Contig4
Cuticle protein 19.8	89.79	55	0.01149462	2.155213952	Unigene44049
Cuticle protein 19-like	77.81	6	0.0120524	2.096478403	CL4340.Contig3

**Table 2:** Cuticle proteins and homologies in U73122-treated *A. lucorum*.

Protein	Blast similarity mean (%)	Peptides (95%)	*p*-value	Ratio (average)	Accession
Cuticle protein 7	83.01	46	0.008437019	0.481294185	CL4657.Contig1
Cuticle protein 10.9-like	77.66	3	0.018015705	0.427869186	Unigene44908
Cuticle protein 21-like	97.29	569	1.97353E-05	0.405525729	Unigene12071
Cuticle protein 21-like	92.38	71	0.003097548	0.297643058	Unigene41367
Larval cuticle protein A2B-like	95.32	487	0.016687083	0.273016788	CL1002.Contig1
Larval cuticle protein A2B-like	93.72	548	0.00033265	0.272292286	Unigene44898
Cuticle protein 16.5-like	68.54	374	0.016726727	0.224266387	Unigene10138
Cuticle protein 8-like	90.16	57	0.010342484	0.213135041	Unigene19243
Cuticle protein 3	83.07	14	0.001466315	0.184845738	Unigene13702
Cuticle protein 19.8	89.79	55	0.009174642	0.184463348	Unigene44049
Cuticle protein 70, isoforms A and B	100	40	0.007508727	0.140011718	Unigene5243
Larval cuticle protein A2B-like	94.89	608	0.001391079	0.094018778	Unigene44897

**Table 3:** Cuticle proteins and homologies in 20 + U73122-treated *A. lucorum*.

Protein	Blast similarity mean (%)	Peptides (95%)	*p*-value	Ratio (average)	Accession
Cuticle protein 2-like	58.99	2	8.47802E-06	0.472068041	Unigene2357
Cuticle protein 16.5-like	69.61	374	0.02345216	0.432273552	Unigene10138
Larval cuticle protein A2B-like	71.48	487	0.004508908	0.41866003	CL1002.Contig1
Cuticle protein 16.5	66.30	57	0.037728864	0.385975547	Unigene23777
Larval cuticle protein A2B-like	71.65	80	0.001128939	0.352907077	CL1002.Contig2_
Larval cuticle protein A3A-like	67.08	380	0.027402238	0.352296807	Unigene19994
Cuticle protein 16.5	67.72	13	0.000400274	0.305423185	Unigene1414

**Figure 1:**
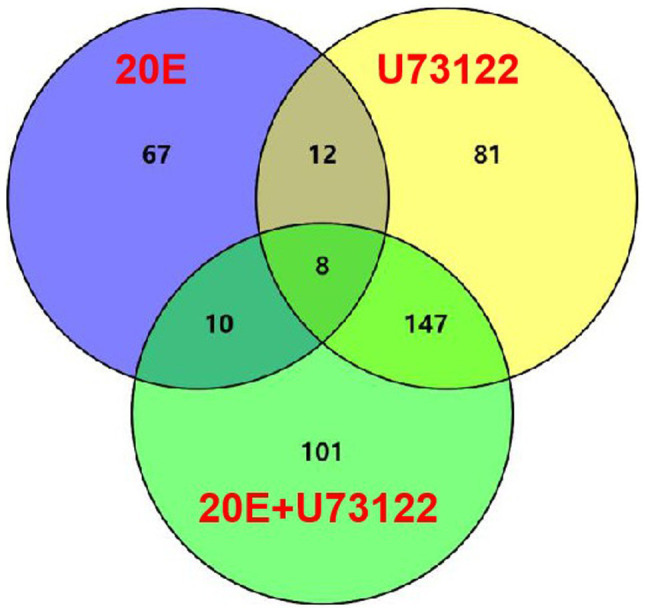
Differentially expressed proteins (DEPs) with a change ≥2.0-fold in *Apolygus lucorum* after treatment with 20E, U73122, and 20E + U73122, respectively. Coregulated DEPs between two time points are also determined.

Venn diagram (Figure 1; Supplementary Table S3) showed that only eight DEPs were commonly shared among all three groups. Four proteins, including pathogenesis-related protein 5-like, cuticle protein 19.8, trans-sialidase, and larval cuticle protein A2B-like, were upregulated after 20E treatment but downregulated in both the U73122/control and 20E + U73122/control groups. Meanwhile, cathepsin L1, protein takeout (hemolymph juvenile hormone binding protein), ATP-dependent RNA helicase p62-like, and myosin-9 isoform X1 were all downregulated.

### GO Analysis of Differentially Expressed Proteins

**Figure 2:**
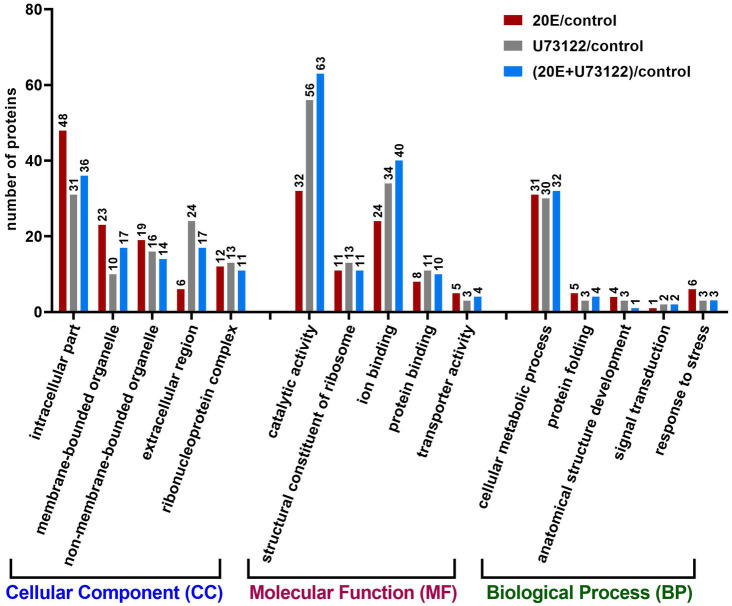
Gene ontology analysis of 611 proteins differentially expressed after treatment with 20E, U73122, and 20E + U73122 in *A. lucorum*, respectively. Proteins are annotated by biological process, cellular component, and molecular function.

Since no complete reference proteome database of *A. lucorum* was available, we annotated the identified DEPs by Blast2GO suite in the three groups. There were 235 DEPs after 20E treatments, 252 DEPs after U7312 treatment2, and 265 DEPs after 20E + U72122 treatment. The assigned GO terms/numbers represented their functional relevance in the categories of cellular component (CC), molecular function (MF), and biological process (BP). In terms of function, there were 94–108 significantly enriched GO terms concerning cellular component seen after different treatments, in which the top significant GO terms were intracellular part, the membrane-bounded organelle, and extracellular region (Figure 2). Intriguingly, cellular component GO term categories were found exclusively after 20E treatment, enriched in intracellular part, membrane-bounded organelle, and non-membrane-bounded organelle compared with U73122 and U73122 + 20E treatments. The top-listed GO terms were related to catalytic activity, ion binding, and protein binding with respect to molecular function. There were more DEPs enriched in catalytic activity, ion binding, and protein binding after U73122 and U73122 + 20E treatments, which were decreased after 20E treatment. There were only 40–50 DEPs enriched in GO term in the biological process category, with no significant differences among the three treatment groups.

### KEGG Pathway Enrichment Analysis of DEPs

**Figure 3:**
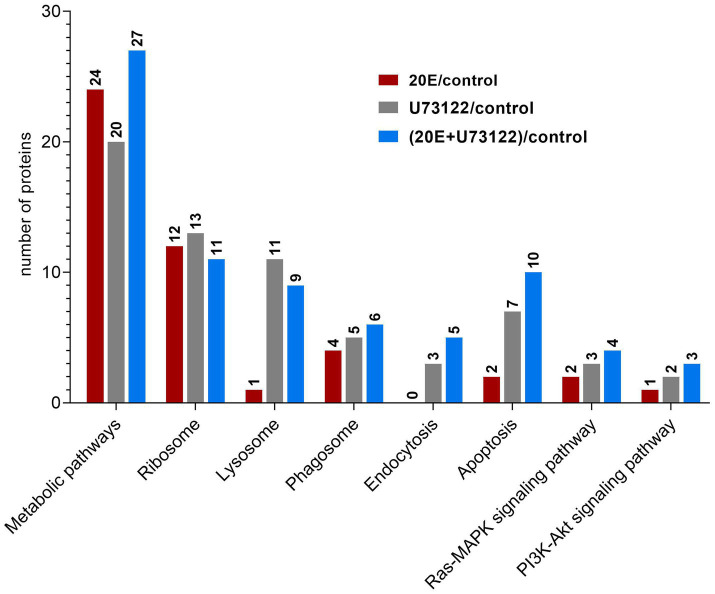
Kyoto Encyclopedia of Genes and Genomes (KEGG) pathway analysis of DEPs identified after treatment of *A. lucorum* with 20E, U73122, and 20E + U73122.

**Figure 4:**
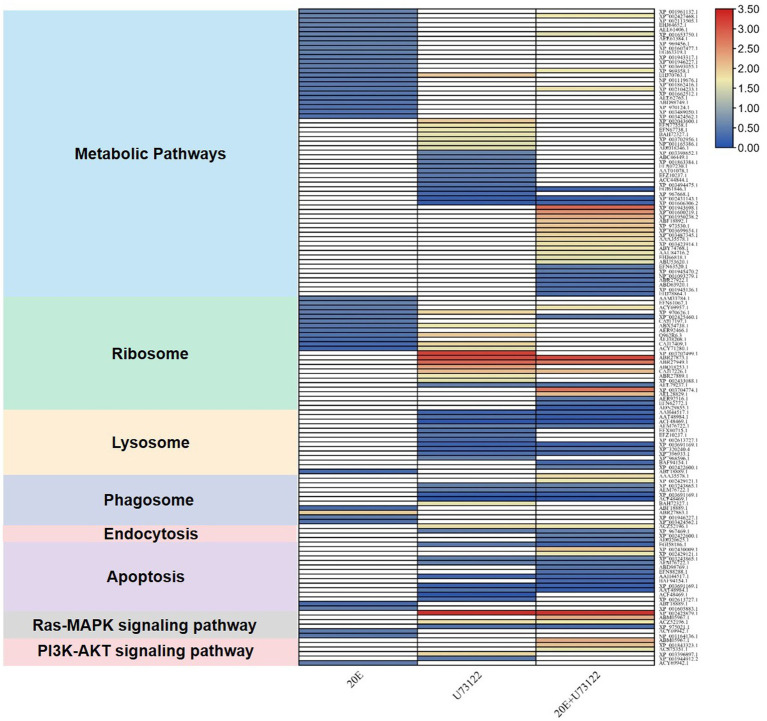
Enrichment analysis of DEGs in the KEGG pathway after treating *A. lucorum* with 20E, U73122, and 20E + U73122.

“Zoomed-in” KEGG pathway analysis revealed that the identified DEPs were integrated into diverse signaling pathways (Figure 3; Supplementary Table S4). The most significant DEPs among the three groups were associated with metabolic pathways. Comparative analysis showed no overlapping DEPs among the three groups. All DEPs (*n* = 24) in the 20E/control group were downregulated, while both up-and downregulated DEPs were found in the U73122/control (eight upregulated and 12 downregulated) and 20E + U73122/control group (17 upregulated and 10 downregulated). Interestingly, multiple ribosomal proteins were identified as DEPs involved in the ribosome process/pathway. In the 20E/control group, all ribosomal proteins (*n* = 11) were downregulated, while 11 out of 13 DEPs were upregulated in the U73122/control group. In the 20E + U73122/control group, the numbers of up- and downregulated ribosomal proteins were almost even (6:5), suggesting that 20E and U73122 treatments played opposite roles in regulating protein translation (Figure 4).

In contrast to the 20E/control group, DEPs in the U73122/control and 20E + U73122/control groups were more enriched in the processes/pathways of the lysosome, phagosome, endocytosis, phagosome (phagocytosis), endosome (endocytosis), and lysosome. A total of 20 non-redundant DEPs in the U73122/control and 20E + U73122/control groups, including 11 shared ones, were involved in these processes. Most of them (16 out of 20) were downregulated, whereas only 4 DEPs (one upregulated and three downregulated) in the 20E/control group were involved in these processes/pathways. Furthermore, several subunits (A, C, G, and E) of V-type proton ATPase (ATPeV) were identified as DEPs belonging to the phagosome group. Subunits A (catalytic unit) and G, identified in the 20E + U73122/control and U73122/control groups, were upregulated, while subunits C and E (assembling the peripheral stalk to insert the complex into the membrane) were downregulated in the 20E/control group.

### Validation of DEPs

**Figure 5:**
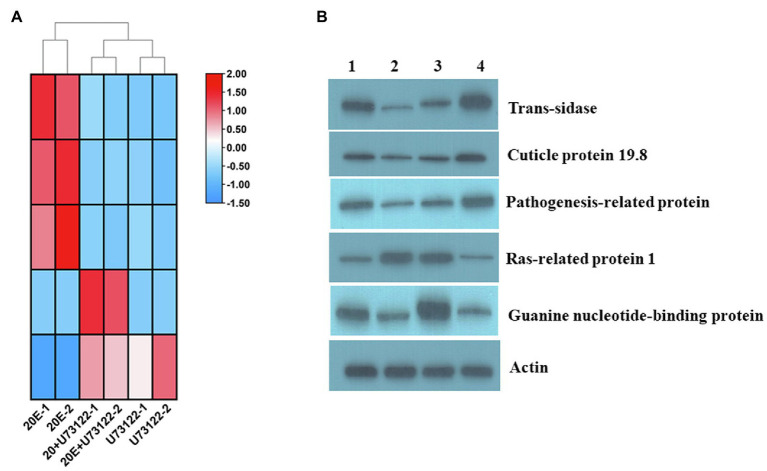
**(A)** Heat map and **(B)** Western blotting of Ras-related protein 1 (Rap1), cuticle protein 19.8, guanine nucleotide-binding protein, trans-sialidase and pathogenesis-related protein in different treatment groups of *A. lucorum* in ITRAQ analysis. *Actin* was used for normalization. Lanes 1–4 are CK, U73122, 20E + U73122, and 20E, respectively.

Based on Venn diagram data, trans-sialidase, cuticle protein 19.8, pathogenesis-related protein, guanine nucleotide-binding protein, and Ras-related protein 1 were selected from different functional categories and gene families to prevent the influence of co-regulation and validate the proteomic data. Western blotting analysis showed that trans-sialidase, cuticle protein, and pathogenesis-related protein were upregulated by 20E, and downregulated by U73122 and 20E + U73122, respectively. On the other hand, Ras-related protein 1 was upregulated (Figure 5) in response to U73122 and 20E + U73122 treatments but downregulated by 20E. Guanine nucleotide-binding protein was clearly upregulated by 20E + U73122, while treatment with 20E and U73122 showed no significant effects on its expression. Results of the Western blots confirmed the above proteomic findings.

## Discussion

Ecdysteroids represent a group of steroid hormones regulating developmental, reproductive, and other critical biological events in insects, and 20E is considered the major ecdysteroid in most insects (Spindler-Barth and Spindeler, 2000; Lafont et al., 2005). This hormone functions by activating target genes in a stage-specific and tissue-specific manner. The combination of two-dimensional polyacrylamide gel electrophoresis (2-DE), MS, peptide sequencing, and database search has been a great tool for determining DEPs, but is relatively complex and unsuitable for quantitating proteins in minute quantities. iTRAQ, a novel stable isotope technique for protein measurement by employing mass spectrometry (Zieske, 2006; Pierce et al., 2008), could comparatively assess eight distinct specimens concurrently and examine proteome profile changes caused by various treatments.

In this work, we have characterized 1,624 non-redundant proteins with two or more unique peptides that were identified by the iTRAQ technology. There were 97 (20 upregulated and 77 downregulated), 248 (42 upregulated and 206 downregulated), and 266 (57 upregulated and 209 downregulated) DEPs identified in 20E, U73122 and 20E + U73122 treated *A. lucorum* nymphs, respectively. Besides affecting development, molting, metamorphosis, and reproduction, 20E also regulates metabolic including innate insect immunity. Previous reports demonstrated that 20E induced apoptosis in insect metamorphosis and pathologies. In *B. mori* Bm-12 cells, 20E induced cell cytotoxicity sequentially *via* autophagy and apoptosis (Xie et al., 2016). The induction and repression of immune reactions by 20E are species- and developmental stage-specific. It has been shown that 20E upregulates prophenoloxidase 1 in *Anopheles gambiae* (Ahmed et al., 1999; Muller et al., 1999) and hemolin in the fatbodies of cecropia moth at the diapausing stage (Roxstrom-Lindquist et al., 2005). As shown above, 2, 7, and 10 DEPs were found in the apoptosis pathway in the 20E/control, U73122/control, and 20E + U73122/control groups, respectively. Almost all DEPs among the three groups were downregulated except spectrin alpha (SPTA) and tubulin alpha-1 in the 20E + U73122/control group. SPTA, a cytoskeletal protein with binding sites for several proteins, is cleaved during apoptosis to alter membrane stability and form apoptotic bodies. Upregulation of SPTA by co-treatment of *A. lucorum* with 20E and U73122 may promote cell survival. Together, the above data indicated that 20E and U73122 negatively regulate apoptosis, and 20E in combination with U73122 might perform a double-edged role in this pathway. Besides positive effects, 20E also suppresses immune response as reported in *Calliphora vicina*, *Bombyx mori*, and *Drosophila*, with downregulated AMPs under high ecdysone condition at the pupal stage, indicating that ecdysone blocks innate immunity (Chernysh et al., 1995; Beckstead et al., 2005; Tian et al., 2010). In the larval molt and during larva to pupa transformation, injected ecdysone induces the downregulation of AMPs, including lebocin-3, gloverin-like protein 2, and nuecin, in the fat bodies of *B. mori* (Tian et al., 2010). The above discrepancies indicate that complex mechanisms may govern the function of ecdysone in innate immunity.

On the other hand, DEPs in the U73122/control and 20E + U73122/control groups were more enriched in the processes/pathways of lysosome, phagosome, and endocytosis. Phagosome (phagocytosis), endosome (endocytosis), and lysosome are inter-crossed organelles for the degradation and recycling of macromolecules (Braun and Niedergang, 2006). A total of 20 non-redundant DEPs in the U73122/control and (20E + U73122)/control groups, including 11 shared ones, were involved in these processes, and almost all these DEPs were downregulated (16 out of 20). In contrast, only 4 DEPs (one upregulated and three downregulated) in the 20E/control group participated in these processes/pathways. These data indicate that PLC predominantly negatively regulates the processes/pathways of phagosome, endosome, and lysosome. PLC is known to contribute to the differentiation/activation of cells controlling both innate and adaptive immunities. PLC also affects tyrosine kinase-associated pathways for inhibiting Stat5 *via* recruitment of the protein tyrosine phosphatase SHP-1, with which PLC and Stat5 interact in the SPS complex. This complex substantially regulates immune cell activation (Kawakami and Xiao, 2013). Furthermore, several subunits (A, C, G, and E) composed of V-type proton ATPase (ATPeV) have been identified as DEPs and classified into the phagosome group. ATPeV is a multi-subunit protein that containing 14 different polypeptides and locates on the organelle membrane. It is involved in acidifying endocytic and phagocytic organelles to regulate ligand release, macromolecule breakdown, and cation uptake. Subunits A (catalytic unit) and G, identified in the 20E + U73122/control and U73122/control groups, respectively, were upregulated, while subunits C and E (assembling the peripheral stalk to insert the complex into the membrane) were downregulated in the 20E/control group, indicating that 20E and U73122 had opposite functions in the regulation of ATPeV activity and the subsequent phagosome process/pathway.

DEPs among the three groups were also found to regulate the Ras-MAPK and PI3K-AKT pathways. In response to stimuli, activated Ras, a small G protein, triggers the MAPK (RAF–MEK–ERK) and PI3K-AKT cascades to control critical cell events, including growth, proliferation, differentiation, migration, and apoptosis (Bonni et al., 1999; Zhang et al., 2014). Ras-related protein 1 (Rap1) was identified as a DEP with increased expression in both the U73122/control and 20E + U73122/control groups, consistent with Western blot results, which indicated that U73122 upregulated rap1. Rap1 was reported to interfere with mitogen-activated protein kinase (MAPK) signaling by trapping the RAF protein. In insects, ecdysteroidogenesis by prothoracic glands (PGs) is controlled by the brain peptide prothoracicotropic hormone (PTTH; Smith and Rybczynski, 2011; Johnson et al., 2013). The mature PTTH interacts with and induces its receptor Torso, a *D. melanogaster* receptor tyrosine kinase (Grillo et al., 2012; Johnson et al., 2013). Induced Torso activates MAPK signaling that encompasses Rap, RAF, MAPK kinase (MEK), and extracellular signal-regulated kinase (ERK), stimulating ecdysteroidogenesis. External U73122 suppressed the expression of Rap1, suggesting that PLC negatively regulated the Ras–RAF–MEK–ERK pathway, which was involved in the biosynthesis of 20E. Furthermore, guanine nucleotide-binding protein G(I)/G(S)/G(T) subunit beta-1 (GNB1) was found to be an upregulated DEP in the 20E + U73122/control group, and Western blot confirmed these results. GNB1 is a subunit of the heterotrimeric G protein. Its principal function is to form a G-beta dimer with the G protein γ subunit (Gγ) to generate a heterotrimeric G protein with G protein α subunit (Gα). Heterotrimeric G protein could sense the signal transferred by GPCR and pass it to downstream molecules. It has been reported that G protein-coupled receptors (GPCRs) contribute to 20E as a membrane receptor in signaling pathways and transfer the signals into the intracellular space to open calcium channels, causing programmed cell death (Oldham and Hamm, 2007). Interestingly, PLC could be stimulated by GNB1 (Camps et al., 1992). In the lepidopteran insect *H. armigera*, 20E regulates calponin phosphorylation and translocation into the nucleus (Liu et al., 2011). In addition, 20E can increase intracellular calcium levels *via* ecdysone-associated GPCR (Cai et al., 2014), or through G protein (Ren et al., 2014). Moreover, 20E controls gene transcription *via* the GPCR, G protein, PLC, calcium, and PKC non-genomic pathway (Liu et al., 2014b). Another ecdysone-responsive GPCR, termed ErGPCR2, also contributes to 20E-induced non-genomic biological events in *H. armigera* (Jing et al., 2015), indicating that 20E cooperates with U73122 to positively regulate GPCR-mediated non-genomic pathway. In contrast, downregulated 14-3-3ζ was identified as a DEP in the 20E/control group. In addition, 14-3-3ζ, the adaptor protein, interacts directly with RAF to serve as a negative regulator of RAF function. Furthermore, 14-3-3ζ also activates PI3K *via* binding of the p85 regulatory subunit, suggesting that 20E exerts different roles in the Ras-MAPK and PI3K-AKT pathways. Taken together, these data showed that 20E and U73122 exert opposite roles in Ras-MAPK and PI3K-AKT regulatory pathways.

Moreover, there were eight DEPs shared in all three groups. Four proteins, including pathogenesis-related protein 5-like, cuticle protein 19.8, trans-sialidase, and larval cuticle protein A2B-like, were upregulated after 20E treatment but downregulated in both the U73122/control and 20E + U73122/control groups. Meanwhile, cathepsin L1, protein takeout (hemolymph juvenile hormone binding protein), ATP-dependent RNA helicase p62-like, and myosin-9 isoform X1 were all downregulated. It indicated that proteins regulated by 20E and PLC were significantly different, in agreement with their characteristics of stimulating distinct cellular responses (Liu et al., 2014b; Chen et al., 2019; Tan et al., 2021).

The cuticle of insects has multiple layers, including the epicuticle, procuticle, and endocuticle, differing in protein profile, structural properties, and physiological roles (Willis, 1996). The properties of insect cuticles depend on the construction of cuticular proteins, and various cuticle types show pronounced differences in mechanical features in association with the characteristics of individual proteins (Andersen, 2011). As shown above, all identified cuticular proteins were upregulated to varying degrees after 20E treatment (Table 1), such as larval cuticle protein A2B-like, pupal cuticle protein C1B-like, cuticle protein 19.8, and cuticle protein 19-like. Therefore, cuticular proteins could be induced by 20E in *A. lucorum*. Mounting evidence have suggested that ecdysone signaling is critical for cuticle biosynthesis and accumulation (Riddiford et al., 2001; Kozlova and Thummel, 2003). The production of cuticular proteins *via* the ecdysone and juvenile hormones is well-known in multiple insects (Charles, 2010). In *Bombyx mori*, several classes of cuticular genes with distinct profiles during development and various ecdysone responses from wing disc have been reported (Zhong et al., 2006; Futahashi et al., 2008). Moreover, the temporal expression of cuticular protein 95 (CPR95) was regulated by the ecdysone-responsive transcription factor E74A in the wing discs of pre-pupa (Wang et al., 2010). Indeed, most cuticular genes are induced by an ecdysteroid pulse, with their expression requiring the existence and suppression of 20E (Zhong et al., 2006; Soares et al., 2007; Nita et al., 2009; Wang et al., 2009a). It is similar to the finding reported at the stage around ecdysis (Noji et al., 2003; Lemoine et al., 2004; Wang et al., 2009b). Remarkably, 12 cuticular proteins and their homologous molecules were downregulated after U73122 treatment, while seven cuticular proteins and their homologous molecules were downregulated by 20E + U73122 (Tables 2 and 3) treatment. Phospholipase C represents a group of enzymes contributing to the cellular turnover of inositol-containing phospholipids (Dai et al., 2015). It has been reported that PLC is involved in 20E signal transduction. In this study, while the addition of exogenous U733122 inhibited PLC activity, it caused suppression of cuticular proteins and their homologous molecules, consistent with Western blot results. Cuticle protein 19.8 was upregulated by 20E, but downregulated upon treatment with U73122 and 20 + U73122. It indicates that PLC may participate in the cuticle protein biosynthesis *via* modulating regulation by ecdysone.

In summary, DEPs were differentially expressed in the 20E/control, U73122/control, and (20E + U73122)/control groups. Functions of these proteins in 20E signaling and downstream responses deserve further attention, such as involvement of cuticular proteins and modulation of immune response in *A. lucorum*. Collectively, these findings provide a more comprehensive understanding of the role of PLC in 20E signal transduction and protein interactions involving PLC under 20E regulation.

## Data Availability Statement

The original contributions presented in the study are included in the article/Supplementary Material, further inquiries can be directed to the corresponding authors.

## Author Contributions

Y-AT, L-BX, and D-JH designed the experiments. Y-AT, X-DZ, JZ, and Q-QJ performed the experiments. Y-AT, X-DZ, and KZ-S wrote the manuscript. All authors contributed to the article and approved the submitted version.

## Funding

This study was supported by the Chinese Agricultural Research System (CARS-20-18), the Jiangsu Agricultural Science and Technology Innovation Fund [CX(21)3088], and the National Natural Science Foundation of China (31301668).

## Conflict of Interest

The authors declare that the research was conducted in the absence of any commercial or financial relationships that could be construed as a potential conflict of interest.

## Publisher’s Note

All claims expressed in this article are solely those of the authors and do not necessarily represent those of their affiliated organizations, or those of the publisher, the editors and the reviewers. Any product that may be evaluated in this article, or claim that may be made by its manufacturer, is not guaranteed or endorsed by the publisher.

## Footnotes

^1^ https://www.blast2go.com/

^2^ http://rsb.info.nih.gov/ij/index.html
